# Bursaries, writing grants and fellowships: a strategy to develop research capacity in primary health care

**DOI:** 10.1186/1471-2296-8-19

**Published:** 2007-04-05

**Authors:** Karin Ried, Elizabeth A Farmer, Kathryn M Weston

**Affiliations:** 1 Primary Health Care Research Evaluation and Development (PHCRED) Program at the Department of General Practice, Flinders University, Adelaide, South Australia, Australia; 2 Discipline of General Practice, The University of Adelaide, Adelaide SA 5005, Australia

## Abstract

**Background:**

General practitioners and other primary health care professionals are often the first point of contact for patients requiring health care. Identifying, understanding and linking current evidence to best practice can be challenging and requires at least a basic understanding of research principles and methodologies. However, not all primary health care professionals are trained in research or have research experience. With the aim of enhancing research skills and developing a research culture in primary health care, University Departments of General Practice and Rural Health have been supported since 2000 by the Australian Government funded 'Primary Health Care Research Evaluation and Development (PHCRED) Strategy'.

A small grant funding scheme to support primary health care practitioners was implemented through the PHCRED program at Flinders University in South Australia between 2002 and 2005. The scheme incorporated academic mentors and three types of funding support: bursaries, writing grants and research fellowships. This article describes outcomes of the funding scheme and contributes to the debate surrounding the effectiveness of funding schemes as a means of building research capacity.

**Methods:**

Funding recipients who had completed their research were invited to participate in a semi-structured 40-minute telephone interview. Feedback was sought on acquisition of research skills, publication outcomes, development of research capacity, confidence and interest in research, and perception of research. Data were also collected on demographics, research topics, and time needed to complete planned activities.

**Results:**

The funding scheme supported 24 bursaries, 11 writing grants, and three research fellows. Nearly half (47%) of all grant recipients were allied health professionals, followed by general practitioners (21%). The majority (70%) were novice and early career researchers.

Eighty-nine percent of the grant recipients were interviewed. Capacity, confidence, and level of research skills in ten core areas were generally considered to have improved as a result of the award. More than half (53%) had presented their research and 32% had published or submitted an article in a peer-reviewed journal.

**Conclusion:**

A small grant and mentoring scheme through a University Department can effectively enhance research skills, confidence, output, and interest in research of primary health care practitioners.

## Background

Research and research literacy play an increasingly important role in ensuring and enhancing the provision of evidence-based health care. Historically, primary health care professionals have not adequately been trained in research methodology, a deficit which has been recognised internationally [1-4].

In 2000, the Australian Government addressed the need for building research capacity in the primary health care sector by providing 50 million AUD funding over a period of six years for the national 'Primary Health Care Research Evaluation and Development (PHCRED) Strategy – Phase One' [5]. Regional PHCRED programs at University Departments of General Practice and Rural Health formed one part of this Strategy [5]. The PHCRED program at Flinders University in South Australia developed a variety of research capacity building activities under the umbrella of a newly formed South Australian Research Network for primary health care called 'SARNet' [6,7].

In this article, we report the outcomes and evaluation of the research network's grant funding scheme which supported a multidisciplinary cohort of 38 primary health care professionals and early career researchers between 2002 and 2005. Funding was awarded in a number of ways: as a research bursary ($5,000) to develop and undertake a small research study, as a writing grant ($500) to encourage the dissemination of research findings in peer-reviewed journals, or as research fellow position (0.2–0.5 FTE over 1 year) to support research skills development in an academic environment. Each researcher was mentored by a member of the PHCRED core team of four part-time academics and had access to SARNet network activities including training workshops, web-based educational material, an online discussion forum and other network events.

There is a paucity of empirical studies systematically evaluating capacity building programs, which can provide valuable insights into impact, efficiency and effectiveness and help plan future initiatives [3]. Our study addresses this gap and provides an example of a successful strategy for building research capacity in primary health care.

## Methods

With the aim of building research experience, skills and confidence of primary health care professionals, the Flinders PHCRED program launched the bursary and writing grant scheme in 2002. Calls for applications were announced annually via the research network 'SARNet' website [8] and the network's member list [7]. Professionals in general practice, allied health, and other areas of primary health care were eligible to apply for PHCRED funding. In addition to the bursary and writing grant holders, the PHCRED program supported a small number of research fellows. Research fellows were included in the evaluation of the PHCRED funding scheme, as they had access to the same mentoring team and network resources as the other funding recipients.

Researchers were assigned a designated mentor, who provided continuity of expertise, advice and support at all stages of the research project, including where necessary the development of a research plan, submission of ethics applications, advice on data collection and analysis, and preparation of a study report or article for peer-reviewed publication. Regular meetings between mentor and mentee were arranged over the course of the project, taking place either face-to-face, via teleconferences, or through email correspondence. Occasionally guidance in specific areas, such as statistics and consumer issues, was sought from external experts. Mentoring embraced adult learning principles: giving support when and where needed, at the appropriate professional level, and being purpose driven. Mentoring time varied according to level of expertise of the researchers and the nature of the research project.

All funding holders were required to present their work at a suitable event (e.g. conference), and write a comprehensive final report. Initially, grants were provided for the duration of one year. In some cases, time frames of one year proved to be insufficient and needed to be extended to achieve project objectives.

To evaluate the funding scheme, 38 PHCRED funding holders who had completed their projects by early 2006 were invited to participate in a semi-structured 40 minute telephone interview conducted by an external and independent interviewer. Feedback was sought on acquisition of research skills, publication outcomes, the impact on confidence, and interest in pursuing research in the future in relation to the PHCRED funded project. Answers were recorded verbatim, and participants checked the transcripts for correctness. We analysed quantitative data using the statistical program SPSS 13.0, and employed a phenomenological approach to code qualitative data according to themes. Ethics approval was obtained by the Social and Behavioural Research Ethics Committee at Flinders University.

While data on basic demographics, profession, research topic and outcomes were available for all grant holders (n = 38), other findings are based on participants' feedback in our evaluation study (n = 34). Grants supported individuals but also research teams, for example, a consumer organisation of seven members, and nursing student groups of 20 members were recipients of single grants. To evaluate the impact of bursaries on these research teams, we asked a representative senior member of the group to give feedback on behalf of their team.

## Results

### Demographics and research activities

Thirty-four (89%) grant holders participated in the evaluation; 21 of 24 bursary holders (88%), 10 of 11 writing grant holders (91%), and 3 of 3 research fellows. The majority of Flinders PHCRED funding recipients lived in metropolitan Adelaide (73.5%), were female (74%), and between 35 and 54 years of age (79%). Nearly 20% of the grant recipients were based in rural South Australia and Victoria, in the Greater Green Triangle (GGT) region [9]. The PHCRED programs at Flinders University in Adelaide (SA) and the Greater Green Triangle University of Rural Health in Warrnambool (VIC) collaborated on SARNet related activities and adopted the Flinders PHCRED bursary and writing grant scheme in 2004. All bursary and writing grant holders included in this study were mentored by the same PHCRED core team of four academics. Meetings were held either face-to-face or by email and telephone depending on time schedules, location, and project status.

**Figure 1:**
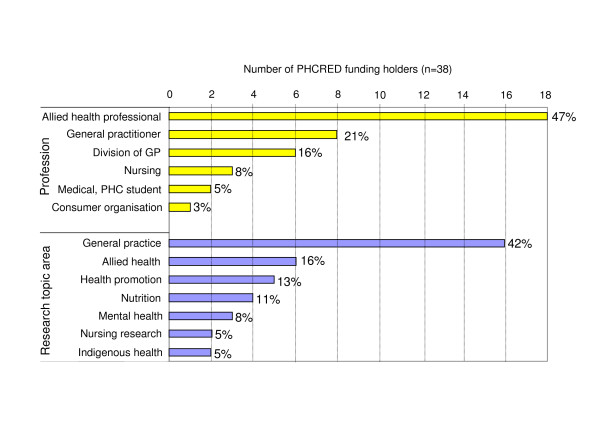
**Grant holders' professions and research topic areas**. The graph provides an overview of all Flinders PHCRED funding holders' (n = 38) professions (yellow) and research topic areas (blue) investigated between 2002 and 2005. Nearly half of the funding holders were allied health professionals (47%), followed by 21% of general practitioners, and other primary health care professionals. Research projects undertaken covered general practice topics (42%), and allied health topics (34%) including nutrition and mental health.

Most of the grant holders were allied health professionals (47%). A smaller number were general practitioners (GPs) (21%), Division of General Practice staff (16%), and nursing professionals, medical and primary health care students, and one consumer organisation (Figure 1). The majority of individual grant holders held at least one postgraduate qualification, including Graduate Diploma (n = 5), Masters Degree (n = 11), and PhD (n = 5). Four grant holders held a Fellowship of the Royal Australian College of General Practice (FRACGP). Research topics included general practice (42%), allied health subjects including nutrition and mental health topics (34%), health promotion, nursing research, and Indigenous health (Figure 1).

**Figure 2:**
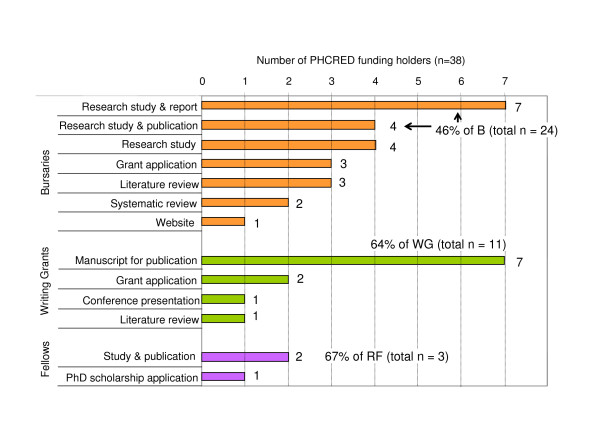
**Main outcomes of grant activities**. The main outcomes of all bursary holders' (orange, n = 24), writing grant holders' (green, n = 11), and research fellows' (purple, n = 3) research projects are summarised. A variety of study designs were supported by the bursaries including a pilot randomised controlled trial, a retrospective case study, focus group research, questionnaire and interview surveys, literature reviews, systematic reviews, and grant applications. Most bursary holders disseminated their findings in a comprehensive report (n = 7), or submitted a manuscript for publication to a peer-reviewed journal (total n = 6: study + publication (n = 4), systematic reviews (n = 2)). Two-thirds (n = 7) of writing grant holders achieved the main purpose of the writing grant, namely the preparation of a manuscript for submission to a peer-reviewed journal. At time of the interview, 2 of the 7 writing grant articles were under review and one paper had been published. All research fellows (n = 3) planned, conducted, analysed their research project. Two fellows had prepared at least one manuscript for peer-reviewed publication and one fellow applied for a PhD scholarship at the end of their positions (0.2–0.5 FTE).

Bursaries supported a wide variety of study designs including a pilot randomised controlled trial, literature reviews, systematic reviews, retrospective case study, grant applications, focus group research, questionnaire and interview surveys, participatory action research, evaluation of health promotion programs, collation and dissemination of health care relevant information via student posters, a website, or a consumer handbook. The majority of writing grants (64%) fulfilled their main purpose supporting early career researchers in the preparation of a manuscript for peer-reviewed publication. All of the three PHCRED funded research fellows made use of their protected time to plan, conduct, and analyse a small research study. Two fellows prepared at least one manuscript for publication, and one fellow decided to continue their research as a PhD student. Figure 2 provides an overview of the achievements of the Flinders PHCRED grant recipients. Some examples of research projects undertaken are given in Table 1.

**Table 1:** Examples of research projects supported by PHCRED funded bursary and writing grants

**Type of funding**	**Examples of research projects**
*Bursaries*	Evaluation of a nutrition intervention at child care centres in South Australia
	Involving consumers in the health system. Support and training need – a consumer perspective
	The 'Food and Move' Project. Promoting healthy eating and physical activity in a secondary school setting
	Well Women's Health Program for Aboriginal and other women living in a remote community
	Effectiveness of non-pharmacological interventions for fatigue: a systematic review
	The effects of Tai Chi exercises on arm lymphoedema and fatigue in women post-mastectomy for breast cancer: a pilot randomised controlled trial
	Stroke patients who aspirate thin liquids – a comparison of current and emerging practice
	Acute Transition Alliance: rehabilitation at the acute/aged care interface
*Writing grants*	Impaired glucose tolerance: GP knowledge, attitudes and practices
	Essential medicine explained: providing medical information to consumers
	Breastfeeding in public: improving community attitudes
	Obesity management in general practice
	After hours presentation in South Australian rural hospitals

### Research experience

**Figure 3:**
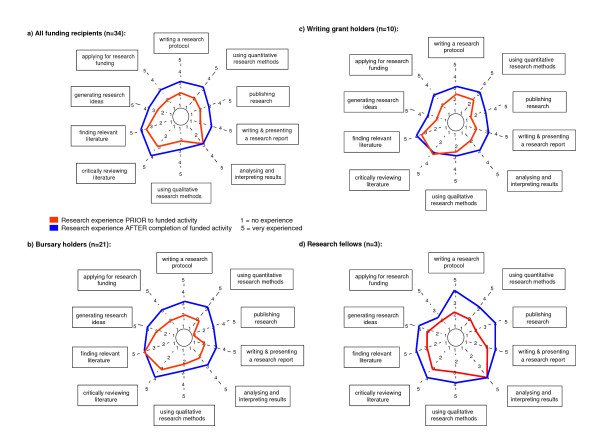
**Median research experience PRIOR and AFTER grant activity, a) of all surveyed funding recipients (n = 34), b) of bursary holders (n = 21), c) of writing grant holders (n = 11), d) of research fellows (n = 3)**. The 'research spider' [7, 10] was used to assess grant holders' research experience before (red) and after (blue) the supported research activity. The level of experience was measured using a five point scale ranging from 1 (no experience) to 5 (very experienced). The median research skill levels of all surveyed funding recipients (n = 34) increased for 9 out 10 skill areas (a). Research skill development by category of grant funding is shown for bursary holders (b), writing grant holders (c), and research fellows (d). Writing skills increased by up to 2 points in all categories.

We evaluated the impact of the grant funding scheme on skills development using a visual tool featuring ten core research skill areas, the 'research spider' [7,10]. A copy of the research spider was available to grant holders during the telephone interview. Grant holders were asked to rate their perceived skill level from 1 = 'no experience' to 5 = 'very experienced' prior to and after their research activity. Figure 3a summarises the median research skill levels at the two time points of all PHCRED funding recipients participating in the evaluation (n = 34). Research skills levels increased in 9 out of 10 skill areas, including writing for publication and use of quantitative and qualitative research methods.

Figures 3b–d depict research skills development by category of grant funding (bursary, writing grant, research fellow). The greatest impact across all categories was achieved in academic writing activities, in particular in 'publishing research' indicated by a 1.5–2 out of 5 point increase in all three groups. The median score after the grant activity was 3 ± 1.1 for the bursary and writing grant recipients and 4 ± 1.1 for the research fellows. Grant activities had little impact on perceived experience levels in literature searching of bursary holders (median score = 4 ± 0.8), critical appraisal skills of writing grant holders (median score = 3 ± 0.7), and on skills for analysing and interpreting results of research fellows (median score = 4 ± 0.6).

To assess the level of prior research experience of grant holders, we asked participants to decide which one of four research categories they considered they belonged. The four research categories formed part of the Flinders PHCRED capacity building model, previously described by Farmer & Weston [6]. The four categories are: non-participants (having little or no previous experience in research); participants (experience as part of a research team); managers/trainers (either leading research, or in formal training to do so); and academics (with, or leading toward a doctorate). Nearly a third of all grant holders (29.4%) rated themselves as novice researchers (non-participants) prior to the funded, and 40% felt they belonged to the 'participant category'. At the end of the funded activity, 35% of the grant recipients considered themselves to have moved to a higher category of research experience, in particular the 'non-participants' of whom 60% felt they have moved from 'non-participant' to 'participant' (Table 2).

**Table 2:** Grant recipients' research categories before and after the funding activity

			Number at each category **after **funding activity			
			
			# 1: Non-participants	# 2: Participants	# 3: Managers/trainers	# 4: Academics
Number at each category **prior to **funding activity	# 1: Non-participants	10	4	5	1	-
	# 2: Participants	13	-	8	4	1
	# 3: Managers/trainers	8	-	-	7	1
	# 4: Academics	3	-	-	-	3

		Total	4	13	12	5

### Capacity, confidence and interest in research

**Figure 4:**
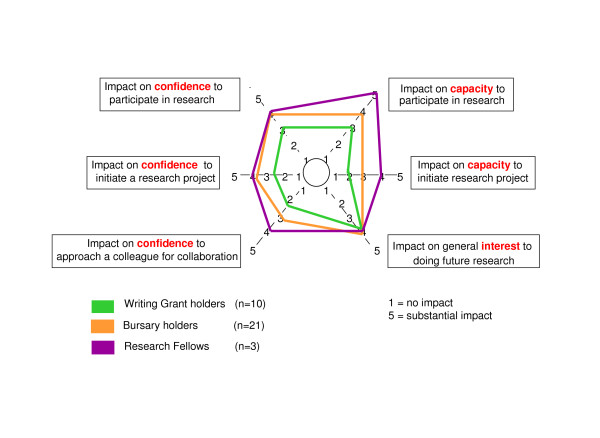
**Impact of grant activity on funding holders' capacity, confidence and interest in pursuing research**. Median impact scores of the supported research activities on participants' capacity, confidence and interest are shown by type of funding, writing grants (green), bursaries (orange), and researcher fellows (purple). The level of impact was measured using a five point scale ranging from 1 (no impact) to 5 (substantial impact). Median impact levels correlated directly to type of funding across all areas, with the writing grants' ($500) impact rating lowest, bursaries ($5,000) in-between, and research fellow positions (0.2–0.5 FTE over 1 year) rating highest. All grant holders indicated that the funding scheme had high impact on their interest to pursue research in the future (median = 4 ± 1.5).

Grant recipients were asked to rate the impact of the supported activity on their capacity and confidence to participate in or initiate a research project, their confidence to seek collegial support for research collaboration, and their general interest in doing future research. Impact was measured on a 5-point scale, with a score of 1 reflecting 'no impact', and a score of 5 reflecting 'substantial impact'. Figure 4 summarises the median impact of the supported research activity on participants' capacity, confidence and interest for each group of grant recipients. Impact scores were directly correlated to the type of funding, with writing grants rating lowest, and research fellow positions rating highest in capacity and confidence issues. Overall, the impact of grant activities on 'capacity to participate in research' was rated highest with a median score of 3 ± 1.1 for writing grant holders, 4 ± 0.9 for bursary holders, and a median of 5 ± 2.3 for research fellows. Somewhat expectedly, writing grants had little impact on building capacity (median = 2 ± 0.9) and confidence (median = 2.5 ± 1.2) to 'initiate a research project'. All grant recipients indicated that the funding scheme had high impact on their 'interest to pursue research in the future' (median = 4 ± 1.5).

### Dissemination of research findings

Nearly two-thirds (62%) of the grant recipients reported to have disseminated their research findings at completion of their grant activity. About half (53%) presented their findings at one or more conferences, including conferences at a state, national, or international level and a third of grant holders had given a seminar to a local audience. Four papers had been published at time of the interview (4 grant recipients) and an additional seven grant recipients had submitted an article for publication in a peer-reviewed journal. Other forms of dissemination included local poster displays, articles in national newsletters, and links to full reports on websites. More than 70% of interviewees were satisfied to have met their goals of the funded activity.

### Aspects of support in the PHCRED funding scheme

In order to assess the support received by the recipients of the Flinders PHCRED funding scheme for capacity building, participants were asked to rate the importance of the following six aspects: funding, access to an assigned PHCRED mentor, external mentoring or supervision, PHCRED team (excluding mentor), access to SARNet web-based research resources, and networking opportunities. Importance was rated on a 5-point scale with a score of 1 indicating this aspect was 'very unimportant' to 5 indicating it was 'very important'. Table 3 provides an overview of the respondents' ratings of which aspects of support were considered 'important' or 'very important'. Receipt of funding for the research project played an important role for most grant holders (85%), while two-thirds indicated that the academic support team (mentor and PHCRED team) was crucial in undertaking their research. In addition, 47% commented that flexibility and support of their workplace were essential in achieving their project's goal.

**Table 3:** Importance of PHCRED funding scheme support to grant recipients (n = 34)

**Support**	**Number (percentage) of grant recipients indicating 'important' or 'very important' n (%)**
Funding	29 (85)
PHCRED mentor	22 (65)
External mentor/supervisor	19 (56)
PHCRED team (excluding mentor)	19 (66)
SARNet web-based research resources	9 (37)
Networking opportunities	12 (35)

Percentages refer to valid responses only. Not all support mechanisms were applicable for all grant recipients.

### Time span for completion of grant activities

**Figure 5:**
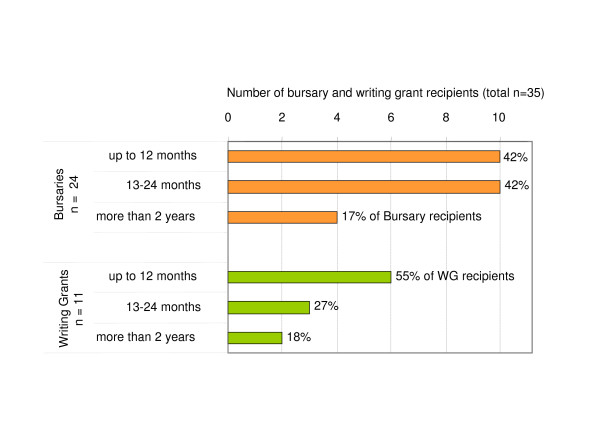
**Time span for completion of bursary and writing grant activities**. Time taken from award of the bursaries (orange) and writing grants (green) to completion of the funded activity are summarised for all of Flinders PHCRED bursary and writing grant holders (n = 35) supported between 2002 and 2005. Overall, the majority of bursary holders (84%) and writing grant holders (82%) completed their projects within a two year time frame.

Due to the one year funding cycles pre-determined by the national funding body, the Australian Government Department of Health and Ageing, all Flinders PHCRED grants were initially awarded for a maximum of one year. However, during the 2002–05 funding period it became apparent that some bursary and writing grant recipients required more than one year to complete and publish their project (Figure 5). The extra time was needed for data collection, analysis, and publication, and project activities were sometimes deferred due to other work commitments (personal communication). Since the awarded funds were of a fixed amount, the mentoring team supported about half of the bursary and writing grant holders beyond the one year time frame. Overall, the majority of bursary holders (84%) and writing grant holders (82%) completed their projects within two years (Figure 5). A small number of bursary and writing grant projects (n = 6) took up to 29 months to be completed (Figure 5).

### Perception of research, barriers and enablers

The PHCRED team was interested in any change experienced by the grant recipients in their perception of research as a result of the supported activity. Qualitative data analysis revealed that 21% reported they found research now less intimidating, 12% commented that they had gained a better understanding of research processes, 8% noted that the experience had helped them to critically reflect on published research, and 6% welcomed the increased awareness of funding sources for research and grant application processes. The following quotes reflect participants' change in perception of research:

• *"The scheme helped demystify research."*

• *"The scheme made research accessible."*

• *"I'm now aware of what's involved in research."*

• *"Research is a learning process."*

• *"Research is more complicated than I thought. There are so many different ways of approaching a project and it takes time to work out exactly what you want to do and choose the right process to get an outcome."*

• *"Research is important for driving change because it collects the evidence to drive change."*

• *"I learned through critical review process that just because something is published it is not necessarily good research."*

• *"I now appreciate the value of discussions with colleagues when applying for funding."*

When questioned about barriers and enablers to future participation in research, more than half (59%) of participants identified 'time' as a significant barrier, 38% were aware of 'financial constraints' and 12% acknowledged that 'limited support by the workplace' could be detrimental to doing research. Further barriers to research mentioned were 'access to experts, e.g. statisticians', and 'support in writing, e.g. grant application'. The following quotes reflect the views on barriers to research expressed by the grant recipients:

• *"There are always other priorities."*

• *"It's easier to get funding for clinical work. Doctors get clinical loading and other health professionals don't, that's an inequity."*

• *"Stepping across into research activities means a loss in income."*

• *"Research is not core business."*

Common enablers to future research activity of grant recipients were identified as collaboration in research teams, forming partnerships (29%), access to academic mentors (27%), and acquired research skills (24%). One bursary holder was particularly impressed with the benefits of the mentoring concept for early career researchers and initiated a mentoring program for students in their University Discipline (School of Nursing and Midwifery).

### Interest in future research activities

The interest in pursuing future research activities expressed by the grant recipients was very encouraging. Almost all of the participants (94%) indicated an interest in pursuing research, 91% felt encouraged to publish research, 88% were enthused to apply for research grant funding, and 76% wanted to attend further research training Nearly two-thirds were considering undertaking postgraduate studies (Table 4). About one-third (31%) of grant holders viewed themselves as becoming 'clinician researchers' in five years time, and 29% said they would work towards gaining an academic position.

**Table 4:** Interest of grant recipients (n = 34) in future research activities

**Interest in ...**	**Number (percentage) of grant holders indicating they were 'interested' or 'very interested' n (%)**
Doing further research	32 (94)
Publishing research	31 (91)
Applying for grants	30 (88)
Attending further research training	26 (76)
Undertaking postgraduate study (incl. further study)	21 (62)

### Early career researchers recommend funding scheme

Nearly all of the Flinders PHCRED funding holders (94%) agreed they would recommend a capacity building initiative, such as the bursaries and writing grants, to other novice researchers. They felt that the combination of mentoring and some funding support could 'kick start' a career in research by providing protected time and expert advice in a supportive environment. The following comments were made by survey participants:

• *"The funding scheme provides a great opportunity to get your 'toes wet'."*

• *"The combination of resources and support is fantastic for novice researchers."*

• *"It helps you to achieve the goals you've set."*

• *"The scheme is a great way of enabling people to get released to do research."*

• *"It gives a kick start with a safety net for time and support."*

• *"There are few other opportunities of this kind, so the initiative is incredibly important."*

## Discussion

Our study provides evidence that a small grant and mentoring scheme can be an effective means of building research capacity of primary health care professionals.

The small grant funding scheme formed a strategy of the Flinders PHCRED model for capacity building of primary health care practitioners [6]. The model embeds key principles of a supportive research environment into capacity building activities. Protected time, mentoring, access to academic expertise, training, infrastructure through a research network, opportunities for presentation, and feedback on manuscripts for peer-reviewed publication are important components of the capacity building framework [11-14]. Feedback and outcomes of the cohort of grant recipients presented here revealed that the funding and mentoring scheme contributed to an overall increase in core research skills (Figure 3) and had a positive impact on self-perceived capacity, confidence and interest in ongoing research involvement on the majority of grant recipients (Figure 4, Table 4).

While support consisted of instrumental (money, time, university facilities) and mentor-specific components (advice, direction, engagement, feedback) [14], outcomes were also dependent on the grant recipient's individual characteristics, e.g. motivation, commitment, and level of previous research experience. Therefore, the effectiveness of this program needs to be viewed in light of these individual characteristics. Since most of the grant applicants were novice or early career researchers (70%, Table 2) the total number of peer-reviewed publications at the end of the grant scheme cannot necessarily be taken as an objective indicator of the effectiveness of the program. Instead, other indicators such as increase in confidence, methodological skills, and adoption of scholarly habits in a supportive environment, previously identified as important enablers to building research capacity and interest [12,13,15], were included in the assessment for effectiveness. Additionally, we found that the one-year time frame for writing grants and bursaries was often not sufficient to complete the project. Development of research skills and the ability to complete a project is very dependent on the individual and the project being undertaken. Other issues such as existing workload and time commitments also need to be considered. Flexibility in funding arrangements for research is therefore important to accommodate the range of skills and circumstances of primary health care professionals.

While, as expected, not all of the grant recipients were in a position to publish their findings at the end of their funding period, the final publication rate including submissions to peer-reviewed journals in our study (32%, 11 out of 34 over a three year period) is comparable to the publication number achieved by a primary care bursary program in the UK (31%, 6 out of 19 over 5 year period) [16]. Articles accepted for publication included, for example, a systematic review on non-pharmacological management of fatigue, and an article on breastfeeding acceptance in public.

It is important to stress that, while publication of research findings is highly relevant, it is also clear that writing skills and knowledge of the publication process, for example how to deal with reviewers' comments, need time and experience to develop.

Writing grants (500 AUD each) provided through the Flinders PHCRED funding scheme can be one strategy to facilitate guided development of academic writing skills (see also [14]). They provided both a link between the novice writer and a more experienced mentor, and a financial incentive for setting time aside to write. The writing process can further be facilitated with peer-supported writing groups [17] which were also established through the Flinders PHCRED program [18] and accessed by the research fellows.

Besides support by experts and peers, outcomes of grant activities were also dependent on individual motivation and commitment. Grant recipients were regularly contacted by the program manager and/or mentor, and progress was assessed on a six-monthly basis. Awarded funds were allocated in two instalments with one part being paid at the start of the grant activity and the remainder at receipt of a comprehensive final report. Frequent contact between the program hub at the University and the bursary and writing grant recipients off-campus was often essential to successful progression and completion of the projects. Most contacts were made by email and telephone, available to all funding recipients, suggesting little difference in regards to support between local and rural practitioners. Time taken and efforts made to provide ongoing motivation and support to a cohort of grant holders should not be underestimated, and need to be considered for long-term sustainability and continuity of capacity building programs similar to the Flinders PHCRED small grant and mentoring program.

Furthermore, the availability of an adequate supply of academic mentors for ongoing development of promising projects and to support research oriented professionals is crucial. Mentoring has consistently been afforded a high level of importance in research training and development [19-21]. At the same time, it clearly is a significant demand on time as our experience indicates. For example, one mentor of the Flinders PHCRED grant scheme worked an equivalent of 0.1 FTE (or 3–5 hours per week) to regularly support a cohort of three bursary recipients who were novice researchers and three writing grant recipients with limited research background. Based on our experience, the time needed to mentor a novice researcher can be estimated at about one hour per week per mentor-researcher relationship. Thus, in addressing issues of time and availability of mentors it is essential that mentoring is viewed as a key component to development of research skills and afforded a high level of priority amongst established researchers. Possible solutions are embedding a mentor scheme in the strategic plans of governing institutions or the establishment of an external mentor program.

The Flinders PHCRED Program operated on a budget of 230,000 AUD per year, which funded on average eight bursaries (total 40,000 AUD), four writing grants (total 2,000 AUD) and one research fellow (30,000 AUD) annually, and included salaries for a small team of four experienced academics (level B-D, totalling about 1.9 FTE), and an administration assistant (0.1 FTE). The core team provided the infrastructure of the program, including development of the funding scheme and its evaluation, development and delivery of training, educational material, website, and newsletters.

Other tangible outcomes of the grant scheme included individuals and groups who have been able to take advantage of their new skills, confidence and knowledge about research processes by forming research collaborations and building local support networks. At least nine of the Flinders PHCRED bursary recipients (out of 24) and all three research fellows have implemented their research findings in their workplace and/or are currently undertaking further postgraduate research training. Two examples clearly demonstrate the impact of the bursary and fellowship grants in this regard. Firstly, a project promoting healthy eating and physical activity in a secondary school setting resulted in implementation of alternative canteen arrangements to provide healthier food alternatives for children as well as development of a physical education curriculum. A second example was the evaluation of a kindergarten program supporting the development of preschool children. In addition to publication in peer reviewed journals, dissemination of findings to local and national policy makers was undertaken and requests were received to explore implementation of program interstate. Other outcomes include involvement in further postgraduate research training as a result of increased interest in research due to the grant activity, with five of the 38 practitioners in our cohort progressing to higher degree candidature at the time of interview.

Because primary health care is multidisciplinary, professionals of any discipline were eligible for PHCRED funded bursaries, writing grants and research fellowships. Strikingly, the majority of applicants supported by Flinders PHCRED between 2002 and 2005 were allied health professionals, nurses and other non-medical health care professionals, implying a high demand and welcomed research opportunity by non-medical disciplines. On the other hand, the eight general practitioners supported by our program conducted projects of generally less elaborate nature and progress was slower compared to the group of non-medical applicants (details not shown). The disparity between general practice research and other disciplines health has been reported by others [22], suggesting that engagement of general practitioners in research might require an approach different to the scheme applied by the Flinders PHCRED model. Change management approaches, such as described by Langley et al. [23] and practice-based research networks [24-26], have been suggested as alternative ways to engage general practitioners in research [4].

Most Flinders PHCRED bursaries and all fellowships resulted in increased skills and knowledge of the recipient in all aspects of a research cycle, from formulating a research question to dissemination of research findings, on a topic relevant to their own practice. In comparison, change management approaches and practice-based networks often concentrate on practitioner's involvement in selected aspects of the research process, e.g. data collection, on a topic of broad general interest [24]. Many general practitioners under pressure of work may feel unable to embrace opportunities to be more involved in research and the solution to developing a research culture amongst general practitioners may require a longer term approach. A comprehensive and practical program to ensure that GP registrars and medical students are trained in aspects of research may be required so that the next generation of general practitioners are better placed to undertake research or to implement research findings.

This article includes views of funding recipients of the bursary, writing grant and fellowship schemes. We acknowledge that the findings of only three research fellows cannot necessarily be generalised. Nevertheless, inclusion of the fellows in our evaluation of the PHCRED funding scheme provided valuable insights into the potential of a structured fellowship program to form part of a career pathway in research for primary health care practitioners. The Australian Government Department of Health and Ageing has responded to this need through providing ongoing funding for a 'Researcher Development Placement (RDP) Program' – 60,000 AUD for each University Department of General Practice or Rural Health – in Phase Two (2006 to 2009) of the PHCRED Strategy [5].

This paper indicates that a small grant funding scheme can have clear and tangible outcomes in the form of publications, increased skills in undertaking research and developing collaborations and increased confidence. Our study adds to the body of knowledge about the role and effectiveness of such schemes in developing strategies for building research capacity amongst primary health care practitioners.

## Conclusion

A small grant and mentoring scheme situated within a supportive research capacity building environment can provide important pathways to generate research skills, confidence and research aware attitudes amongst practising primary health care professionals with limited research experience. In our study the scheme also stimulated further research involvement and encouraged publication and implementation of findings into practice.

## List of abbreviations

AUD – Australian Dollar

B – Bursary

FTE – Full Time Equivalent

GGT – Greater Green Triangle region (SA, VIC)

GP – General Practitioner

PHC – Primary Health Care

PHCRED – Primary Health Care Research Evaluation & Development program

PhD – Doctor of Philosophy, Higher Degree in Health Sciences

RF – Research Fellow

SA – South Australia

SARNet – South Australian Research Network for primary health care

VIC – Victoria

WG – Writing Grant

## Competing interests

The author(s) declare that they have no competing interests.

## Authors' contributions

All authors (KR, EAF, KMW) conceptualised the study. KR conducted data analysis and prepared the manuscript with contributions by KMW and EAF. All authors approved the final version.

## Pre-publication history

The pre-publication history for this paper can be accessed here:


